# StairNet: visual recognition of stairs for human–robot locomotion

**DOI:** 10.1186/s12938-024-01216-0

**Published:** 2024-02-15

**Authors:** Andrew Garrett Kurbis, Dmytro Kuzmenko, Bogdan Ivanyuk-Skulskiy, Alex Mihailidis, Brokoslaw Laschowski

**Affiliations:** 1 Institute of Biomedical Engineering, University of Toronto, Toronto, Canada; 2 Robotics Institute, University of Toronto, Toronto, Canada; 3 KITE Research Institute, Toronto Rehabilitation Institute, Toronto, Canada; 4 Department of Mathematics, National University of Kyiv-Mohyla Academy, Kyiv, Ukraine; 5 Department of Mechanical and Industrial Engineering, University of Toronto, Toronto, Canada

**Keywords:** Computer vision, Deep learning, Wearable robotics, Prosthetics, Exoskeletons

## Abstract

Human–robot walking with prosthetic legs and exoskeletons, especially over complex terrains, such as stairs, remains a significant challenge. Egocentric vision has the unique potential to detect the walking environment prior to physical interactions, which can improve transitions to and from stairs. This motivated us to develop the StairNet initiative to support the development of new deep learning models for visual perception of real-world stair environments. In this study, we present a comprehensive overview of the StairNet initiative and key research to date. First, we summarize the development of our large-scale data set with over 515,000 manually labeled images. We then provide a summary and detailed comparison of the performances achieved with different algorithms (i.e., 2D and 3D CNN, hybrid CNN and LSTM, and ViT networks), training methods (i.e., supervised learning with and without temporal data, and semi-supervised learning with unlabeled images), and deployment methods (i.e., mobile and embedded computing), using the StairNet data set. Finally, we discuss the challenges and future directions. To date, our StairNet models have consistently achieved high classification accuracy (i.e., up to 98.8%) with different designs, offering trade-offs between model accuracy and size. When deployed on mobile devices with GPU and NPU accelerators, our deep learning models achieved inference speeds up to 2.8 ms. In comparison, when deployed on our custom-designed CPU-powered smart glasses, our models yielded slower inference speeds of 1.5 s, presenting a trade-off between human-centered design and performance. Overall, the results of numerous experiments presented herein provide consistent evidence that StairNet can be an effective platform to develop and study new deep learning models for visual perception of human–robot walking environments, with an emphasis on stair recognition. This research aims to support the development of next-generation vision-based control systems for robotic prosthetic legs, exoskeletons, and other mobility assistive technologies.

## Background

Robotic leg prostheses and exoskeletons can provide locomotor assistance to individuals affected by impairments due to aging and/or physical disabilities [1]. Most control systems for human–robot walking use a hierarchical strategy with high, mid [2], and low [3] level controls. Robotic leg control requires continuous assessment of locomotor states for transitions between different operating modes. Previous high-level controllers relied on mechanical, inertial, and/or electromyographic (EMG) sensors for locomotion mode prediction, which are generally limited to the current state, analogous to walking blind. Inspired by the human vision system [4, 5], egocentric vision can uniquely detect the environment prior to physical interaction and thus aid in smooth and accurate transitions. However, classification of walking terrains such as stairs presents additional challenges because of the complex nature of real-world environments, which can vary significantly in style, material, and geometry. The classification of stairs is particularly important because of the increased risk of severe injury from falls if the environment is misclassified.

Previous vision systems have been developed to recognize stairs for robotic leg control using hand-designed feature extractors [6–10] or automated feature engineering via convolutional neural networks (CNNs) [11, 14–18]. However, these systems have inherent limitations in terms of performance and generalizability to new environments because of suboptimal hand engineering and/or training on relatively small image data sets. Recent studies have significantly expanded the number of labeled images [19] and presented the opportunity to use deep learning models to increase performance and generalizability.

Here, we present a comprehensive overview of the StairNet initiative, which was created to support the development of new deep learning models for visual perception of stair environments for human–robot walking. The initiative emphasizes lightweight and efficient neural networks for onboard real-time deployment on mobile and embedded devices. First, we provide an overview the development of our large-scale data set with over 515,000 manually labeled images [12]. We then summarize and compare key research to date in terms of model development (i.e., different algorithms and training methods [12, 20, 21]) and deployment (i.e., mobile and embedded computing [13, 22]). Finally, we discuss the current challenges and future directions. Building on this work, StairNet aims to support the development of next-generation environment-adaptive control systems for robotic leg prostheses, exoskeletons, and other assistive technologies for human locomotion.

## StairNet dataset

Our StairNet data set contains over 515,000 RGB images, which were manually annotated using class labels for environments encountered during level-ground and stair locomotion. To our knowledge, this data set one of the largest and most diverse data sets of egocentric images of stair environments published to date. We made the data set open source at https://ieee-dataport.org/documents/stairnet-computer-vision-dataset-stair-recognition to support the research community and to allow for direct comparisons between different deep learning models.

We developed the StairNet data set using images from ExoNet [19], captured using a chest-mounted wearable camera (iPhone XS Max) in indoor and outdoor environments. The images were saved at 5 frames/s with a resolution of 1280 × 720 with multiple users with varying heights and camera pitch angles. In our initial study, we found that the ExoNet labels contained many overlapping classes, resulting in limited performance [12]. Therefore, we developed new class definitions to manually re-label the images and increase the precision of the cutoff points between the different walking environments (Table 1). We defined four new classes, including level-ground (LG), level-ground transition to incline stairs (LG–IS), incline stairs (IS), and inclined stairs transition to level-ground (IS–LG). We performed three manual labeling pass-throughs to increase annotation accuracy and precision. We removed images that did not contain either level-ground terrain or incline stairs or had significant camera obstructions. Since our data set is designed for stair recognition, there is no loss of characteristics related to the intended application by removing these images, as any classifications made outside of these classes are considered out of scope and would require additional models for classification.

**Table 1:** Class definitions and cutoff points that we developed and used to manually label the StairNet data set [13]

StairNet class	ExoNet class	Class example	Class description
LG	LG Steady State, LG-Door/Wall		An image that contains a level ground environment where incline stairs are not clearly visible
LG–IS	LG–IS		An image with incline stairs where the horizontal surface area of the bottom step or landing is clearly greater than the surface area of other steps visible in the image (i.e., the surface area or depth is approximately 1.5 × the size of subsequent steps)
IS	IS Steady State, IS-Door/Wall		An image with multiple incline stairs where the horizontal surface area of the top and bottom step or landing is not clearly greater than one another
IS–LG	IS–LG		An image with incline stairs where the horizontal surface area of the top step or landing is clearly greater than that of other steps or landings visible in the image (i.e., the surface area or depth is approximately 1.5 × the size of subsequent steps). For an incline stair to be included in the IS–LG class, the horizontal face of the last step prior to level ground must be visible

Our data set also includes information about the class distribution and definitions. The data set mainly comprises images of level-ground terrain (86% of samples) and incline stairs (9%), with two minority classes, IS–LG and LG–IS, which contain approximately 2% and 3% of the samples, respectively. This imbalance is important to consider when selecting classification and resampling methods. For future model development, we suggest using a video-based train-validation-test split, as outlined in [20]. This method assigns all frames within a video episode (i.e., group of neighboring frames) to a single data set split to prevent data leakage and provide a better estimation of real-world performance and generalizability [23]. Scripts for data splitting and data preprocessing can be found on our GitHub.

We developed and tested a number of deep learning models, and training and deployment methods [12, 13, 20–22] using the StairNet data set to directly evaluate and compare their advantages and disadvantages on a common platform, as subsequently summarized and discussed.

## Deep learning models

### Baseline model

**Fig. 1:**
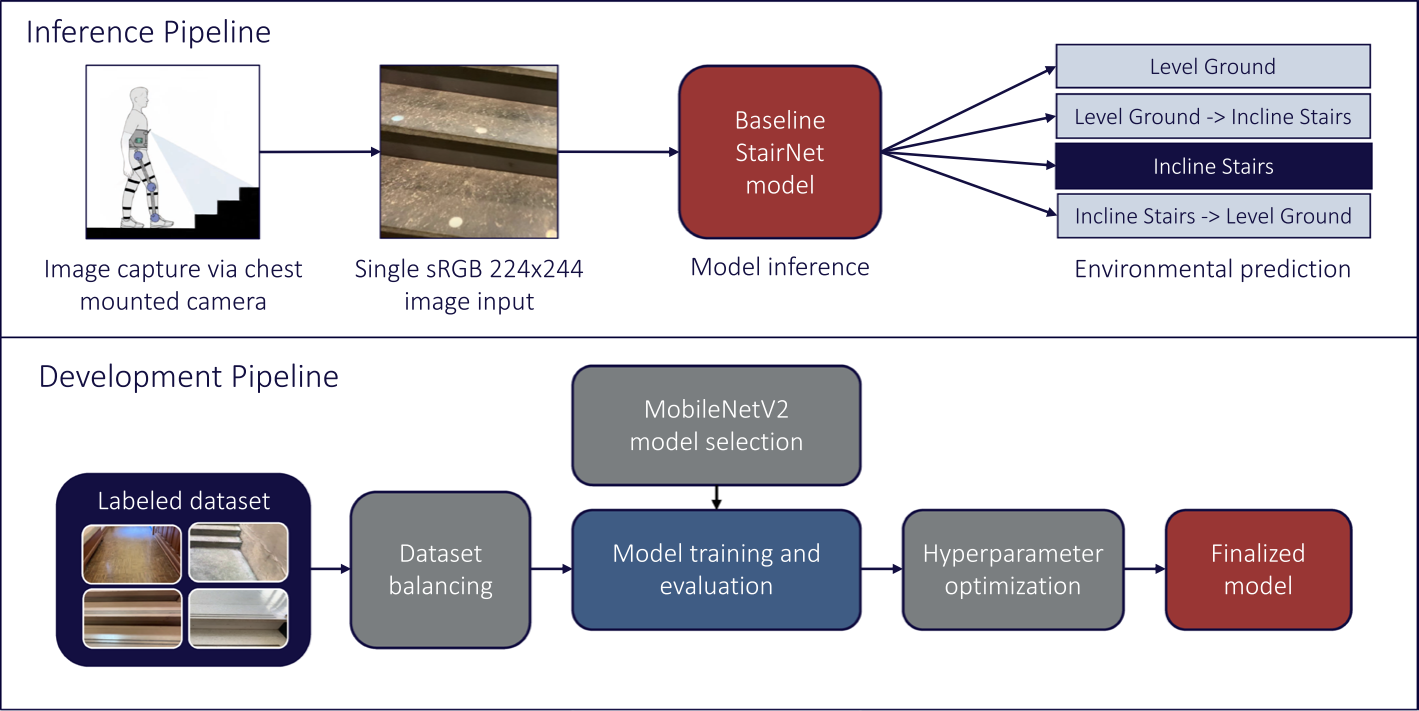
Inference and development pipelines for our baseline StairNet model [12] trained using supervised learning and single images. We developed this model as a reference and benchmark for the other deep learning models presented herein

Our first StairNet model [12] was developed using single-frame supervised learning to provide a baseline reference, as shown in Fig. 1. We developed an efficient 2D CNN based on the architecture of MobileNetV2, which was designed for mobile and embedded vision applications [24, 25]. MobileNetV2’s use of depth-wise separable convolutions with width and resolution multipliers creates a lightweight framework with a trade-off of slightly lower accuracy for significant reductions in computational requirements.

We developed this baseline model using TensorFlow 2.7 [26], starting with the default parameter values from [27]. We used a Google Cloud Tensor Processing Unit (TPU) to efficiently train and evaluate our system. Model variations were evaluated with transfer learning using pretrained weights from ImageNet [28] with various levels of frozen layers (141, 100, 50, 25, 5), randomly initialized weights, regularization via added dropout layers (L2 weight regularization), dropout rates (0.1–0.5) to address overfitting, and oversampling using random resampling and augmentations to address class weight imbalance. We found that transfer learning with five frozen layers and 2.2 million parameters, a dropout rate of 0.2 with no additional dropout layers, and a minimum value of 400,000 images per class (after augmentation and resampling) produced the best accuracy while minimizing the probability of false negatives. Our baseline model underwent a final round of hyperparameter optimization for batch size and learning rate in a high epoch run. After multiple iterations, we finalized the hyperparameters using a reduced base learning rate of 0.00001, a batch size of 128, and a cosine weight decay learning policy. The final model was trained for 100 epochs with early stopping. The model had 2.3 million parameters and 6.1 GFLOPs.

The model was evaluated using the train, validation, and test sets of the “StairNet Dataset”. The model achieved 99.3% and 98.5% accuracies on the training and validation sets, respectively. When evaluated on the test set, the model achieved an overall classification accuracy of 98.4%. In addition, the model achieved an F1 score of 98.4%, weighted precision value of 98.5%, and weighted recall value of 98.4%. The classification accuracy on the test set varied between environments, with categorical accuracies of 99.0% for LG, 91.7% for LG–IS, 96.9% for IS, and 90.5% for IS–LG. The two transition classes (i.e., LG–IS and IS–LG), comprising only 3.1% and 1.8% of the total number of images, respectively, achieved the lowest categorical accuracies. We used this baseline model as a reference and benchmark for the subsequent models that we developed and studied.

### Mobile deployment

To evaluate the real-world performance of our baseline model, we custom-designed a mobile app using TensorFlow Lite (TFLite) [29], Swift 5, and Xcode 13.4.1 [30] for on-device inference [13]. The app prepares images from the camera feed, scaling the input resolution using a square crop to match the input size of our models (i.e., 224 × 224). The model then runs on-device inference, outputting the tensor results in a float-array format containing the confidence values for the four walking environments for each image. The mobile interface displays the output information with the class predictions, along with the onboard inference speed (ms) for the last image.

We used a TFLite interpreter to run the model on the smartphone, which has several advantages over other deployment methods, such as cloud computing. It allows offline execution and inference without requiring an internet connection or communication with a machine learning server while reducing power requirements and privacy concerns as no data is required to leave the device. TFLite also has a small binary size and supports highly efficient models for low inference times, with minimal impact on accuracy during compression.

**Fig. 2:**
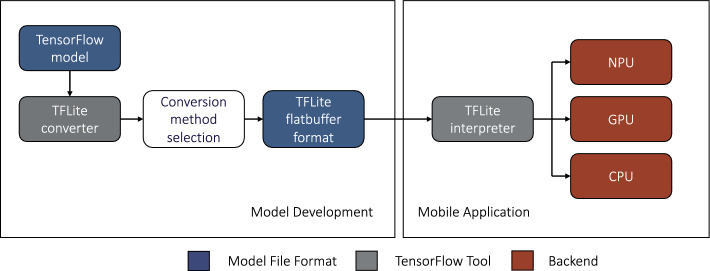
Model conversion and deployment pipeline for our mobile iOS application [13], which we developed to deploy and test our “Baseline Model” for on-device computing

For mobile deployment, our baseline model was converted from its original h5 format to a TFLite flat buffer format. This conversion allows for onboard processing and inference via the on-device interpreter and built-in TFLite infrastructure (see Fig. 2), which supports multiple backend processing options, such as central processing units (CPUs), graphics processing units (GPUs), and neural processing units (NPUs). We experimented with five different conversion methods with varying degrees of compression, which increase inference speed at the expense of accuracy. These compression formats include: (1) float32 compression, (2) post-training float16 quantization, (3) post-training int8 weight quantization, (4) post-training quantization with int16, and (5) post-training int8 full model quantization (i.e., model weights, biases, and activations). Each compression format was evaluated using the StairNet test set to determine its effect on accuracy.

When compressed for mobile deployment, our baseline model had accuracy reductions between 0.001% and 0.111% compared to the full-sized model. The compressed model formats of float32 and float16 quantization resulted in the highest accuracy post-conversion (98.4%). In contrast, the int8 quantization format with both int8 and int16 activations had the lowest post-conversion accuracies of 98.3% and 98.3%, respectively.

We also tested the inference speeds of our baseline model on four different mobile devices (i.e., iPhone 8 + , iPhone X, iPhone 11, and iPhone 13) with four different backend processing options, including a single-threaded CPU, a multithreaded CPU, GPU, and a combination of CPU, GPU, and NPU. An offline test was performed on each device and backend processing option using a pre-recorded video, eliminating variation in camera input on the testing. The pre-recorded video contained stair ascent in indoor and outdoor environments and was loaded on the mobile app to mimic the camera feed. The average inference time was calculated using times sampled at 5-s intervals during the video for each experiment.

The model achieved an inference speed of 2.75 ms on our mobile app using the CoreML delegate and float32 model. The Core ML and Metal delegates, which use parallel processing of CPU, GPU, and NPU, and direct GPU compute, performed best on newer devices, such as the iPhone 11 and iPhone 13. The inference times for these devices were 2.75 ms and 3.58 ms, respectively. In contrast, CPU processing resulted in slower inference times of 9.20 ms and 5.56 ms when using single and multithreaded CPUs. On older devices such as iPhone 8 + and iPhone X, multithreaded CPU achieved faster inference times when compared to single-threaded CPU and GPU processing. When using the CoreML delegate, the float32 compression format delivered the fastest inference speed across all devices. Similarly, the float32 format achieved the fastest inference speeds when running on a GPU with metal delegate. For mobile CPU performance, int8 quantization with int16 model activations resulted in the fastest inference time for single and multithreaded processing, with average speeds of up to 9.20 ms and 5.56 ms, respectively.

In summary, we custom-designed a mobile app to deploy and test our “Baseline Model” using various software and hardware configurations, achieving high classification accuracy and low latency. However, this research was involved standard supervised learning and did not take into consideration the temporal nature of human–robot walking, which motivated the subsequent studies.

### Temporal neural networks

**Fig. 3:**
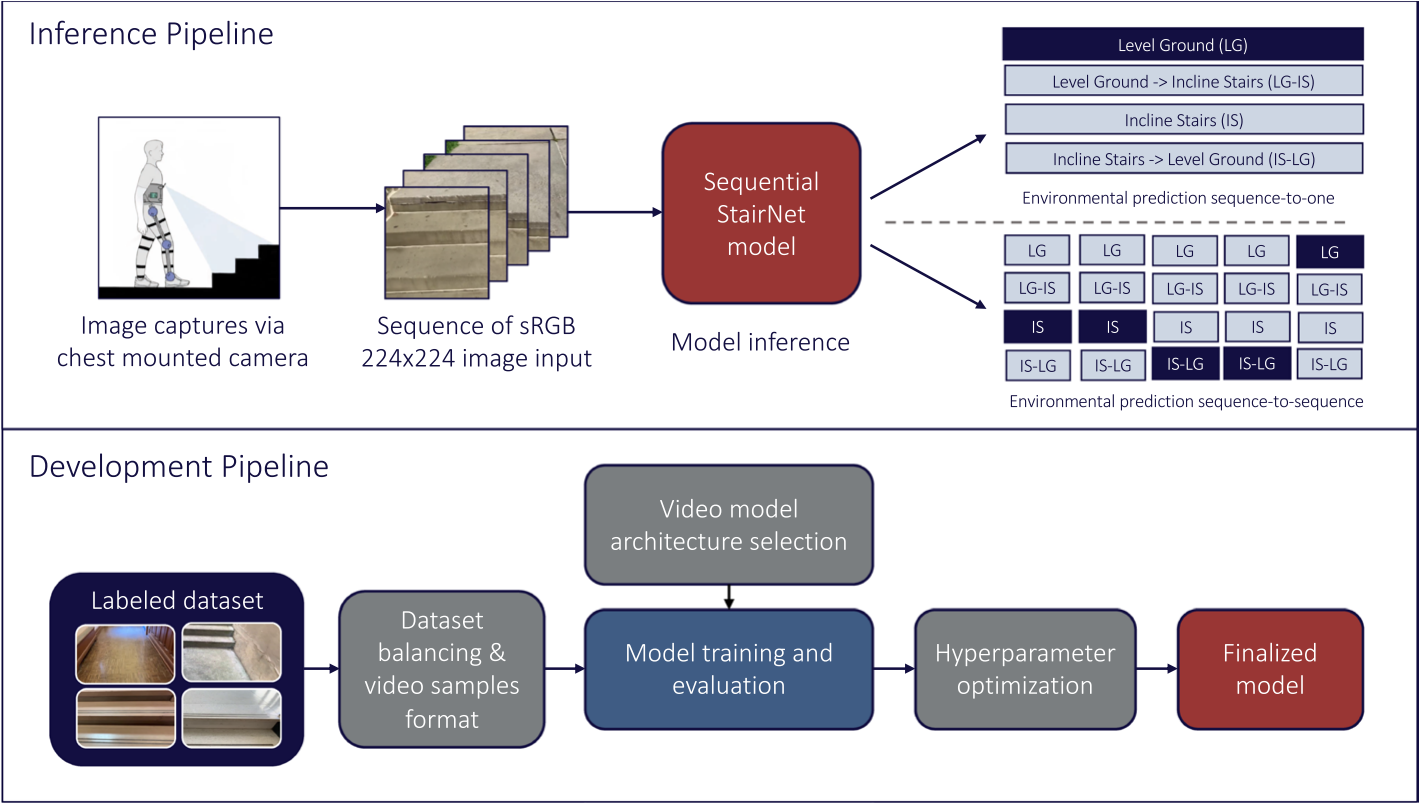
Inference and development pipelines for our temporal StairNet models [20] trained using supervised learning and sequential images. Unlike our previous models that used single image inputs, these temporal neural networks used sequential inputs

To study the effect of sequential inputs on classification performance compared to our baseline model, which used independent frames, we developed a number of state-of-the-art temporal neural networks [20] to exploit information from neighboring frames in the StairNet data set (see Fig. 3). We experimented with different temporal models, including the new lightweight 3D CNN called MoViNet [31], and a number of hybrid encoder architectures, including VGG-19 [32], EfficientNet-B0 [33], MobileNetV2 [25], MobileViT [34], and ViT-B16 [35], each paired with a temporal long–short-term memory (LSTM) backbone [36], and a transformer encoder [37]. We performed focused testing on the 3D MoViNet model, MobileViT with LSTM, and MobileNetV2 with LSTM, which we selected based on their potential to accurately recognize images of stairs and capture temporal dynamics.

First, we experimented with MoViNet, a modified version of MobileNetV3 designed for videos. We used MoViNet's neural architecture search (NAS) to optimize the model parameters, such as the number of layers, convolutional filter width, and number of feature map channels. We adapted the model using a zero-initialized cache feature applied to the boundaries of the video sequences to reduce the growth of model memory, applied 3D convolution to compute the feature maps, and used a stream buffer to reduce the memory use of the model at the expense of a small reduction in accuracy. However, we mitigated this loss in accuracy by using an ensemble of models with two identical MoViNet architectures at a half-frame rate. During inference, the input sequence was fitted to both networks and the mean values of the two models were obtained and passed through the softmax activation function.

We also experimented with MobileNetV2 combined with LSTM. Similar to our “Baseline Model”, the MobileNetV2 architecture was chosen for its efficient model design, optimized for mobile and embedded computing. MobileNetV2 was applied to each frame of the sequence, resulting in a stack of feature maps, which was then fed into an LSTM layer to capture temporal dynamics. The output of the LSTM layer was a sequence of labels for sequence-to-sequence classification or the last predicted label of the LSTM recurrence operation for sequence-to-one classification.

Finally, we experimented with MobileViT, a hybrid encoder model that combines local spatial information from convolutional layers and global information using MobileViT blocks. The model’s convolutional layers projects high-dimensional information encoded using the transformer blocks and projected back to the low-dimensional spaced to be fused with the original feature maps. Similar to MobileNetV2, the MobileViT model was applied to each frame of the sequence. This resulted in a sequence of feature maps, with each map corresponding to one frame. These feature maps were then passed through the transformer layer to capture temporal dynamics of the feature maps of each sequence. In sequence-to-sequence classification, the output of the last transformer block passed through a linear classification head. In sequence-to-one classification, we flattened the transformer layer output before the classification head.

Prior to evaluation, we performed hyperparameter optimization using KerasTuner. The hyperparameter space for each group of models was selected based on the experimental setup and architecture. Once the best hyperparameters were found, each model was trained for 20 epochs using an NVIDIA Tesla V100 32 GB GPU. The Adam optimizer [38] was used with a learning rate of 0.0001, along with a cosine annealing learning rate scheduler.

We used NetScore [39] to compare the models, which balances the classification performance with efficiency and is represented by the following equation:

1 $$\Omega \left(N\right)=20{\text{log}}\frac{acc{\left(N\right)}^{\alpha }}{param{\left(N\right)}^{\beta } flops{\left(N\right)}^{\gamma }}$$

where $acc(N)$ is the classification accuracy (%), $param(N)$ is the number of model parameters, which is indicative of the memory storage requirements, $flops(N)$ is the number of floating point operations, which is indicative of the computational requirements, and $\alpha , \beta , \gamma$ are coefficients that control the influence of each parameter on the NetScore. We assessed the sequence-to-one models by comparing single predictions to their corresponding class label. In contrast, we evaluated the sequence-to-sequence models in two ways. The first method, sequence-to-sequence evaluation, compared a sequence of predictions to a corresponding sequence of labels. The second method compared the anchor frame predictions to the corresponding labels, similar to sequence-to-one.

Of the temporal neural networks that we studied, the 3D MoViNet model achieved the highest classification performance on the StairNet test set, with 98.3% accuracy and an F1-score of 98.2%. The hybrid models with 2D-CNN encoder and temporal blocks (i.e., MobileNetV2 with LSTM and MobileViT with LSTM) struggled to capture inter-frame dependencies with minimal sequences (i.e., five frames per sample) [40] and thus achieved lower classification performance compared to the 3D model. The 3D model had the highest NetScore of 167.4, outperforming the 2D encoder models with scores of 155.0 and 132.1 for MobileViT with LSTM and MobileNetV2 with LSTM, respectively. Our “Baseline Model”, which achieved a NetScore of 186.8, outperformed all the temporal neural networks in terms of efficiency due to its relatively low number of parameters and numerical operations. Finally, we found an increase in performance using sequence-to-one methods on sequence-to-sequence models over the standard sequence-to-sequence method, with an accuracy of 97.3% and 70.7%, respectively, using the same sequence-to-sequence model.

In summary, we found that, of the temporal neural networks that we studied using sequential images, the 3D model outperformed the 2D models with temporal backbones in terms of both image classification accuracy and efficiency (i.e., which takes into consideration the computational and memory storage requirements). We also showed that the 3D model achieved a higher image classification accuracy (98.3%) compared to our 2D “Baseline Model” when retested on the video-based StairNet test set (97.2%). However, the 3D model had a lower NetScore (i.e., less efficient) due to having disproportionally more parameters and operations, which has implications for real-time embedded computing.

### Semi-supervised learning

Compared to the aforementioned research, all of which relied on standard supervised learning, in this section, we studied the use of semi-supervised learning [21] to improve training efficiency by using unlabeled data. The large amounts of publicly available unlabeled data [19] present a viable option to reduce the time and labour-intensive demands required to manually label large-scale data, which was done in the development of the  “StairNet Dataset”. We aimed to show the potential to improve training efficiency by minimizing the number of labeled images while still maintaining comparable performance to our baseline StairNet model.

We used the unlabeled images from the ExoNet data set that were not included in the StairNet data set. However, unlabeled data can present challenges, such as lack of information about class distributions and viability of the images. We performed a visual search of the images and found that the unlabeled data contained images irrelevant to stair recognition and had significant camera obstructions. We used the FixMatch semi-supervised learning algorithm [41] due to its intuitive and feasible implementation compared to more complex algorithms, such as self-training with noise students [42], meta-pseudo-labels [43], AdaMatch [44], and contrastive learning for visual representation [45]. We considered FixMatch a good starting point, although we encourage future research exploring other algorithms.

**Fig. 4:**
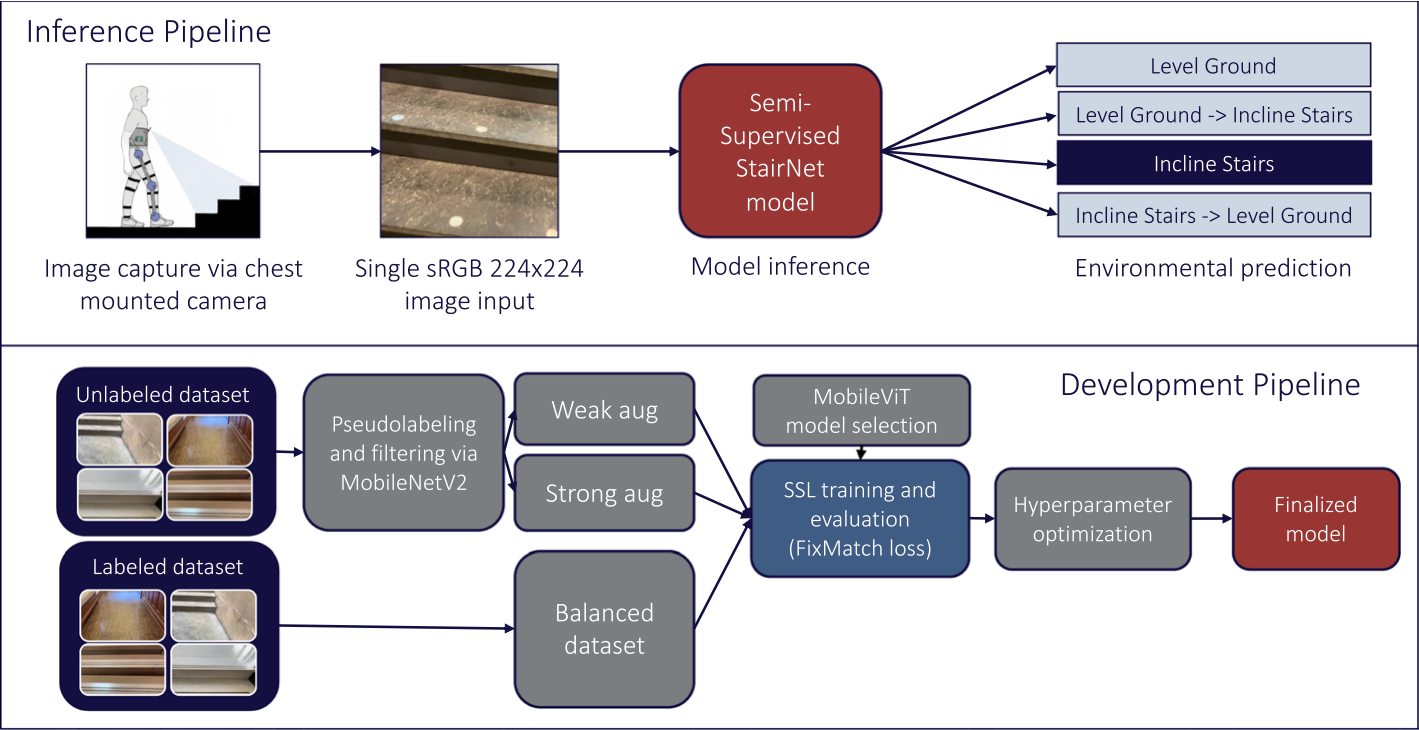
Inference and development pipelines for our semi-supervised learning StairNet model [21] trained using labeled and unlabeled images. Unlike the aforementioned models, this model used large amounts of unlabeled data to minimize the number of required labelled images while still maintaining classification accuracy, therein improving training efficiency

Our semi-supervised pipeline consisted of three major steps (Fig. 4) (1) the labeled images were loaded and oversampled with augmentations, to reduce false positives in training; the unlabeled image logits were retrieved using a supervised pretrained model, from which the pseudo-labels were selected if they surpassed the cutoff parameter τ, (2) weak augmentations (i.e., horizontal flips) and strong augmentations (i.e., color intensity, saturation, small rotations, and horizontal flips) were applied to the unlabeled images, and (3) the MobileViT models were trained using a combination of a supervised loss (i.e., cross-entropy loss) and unsupervised loss (i.e., cross-entropy loss of the inferred weakly augmented images calculated against strongly augmented images). The weight of the unsupervised loss on training was adjusted using the parameter λ. The batch size ratio parameter μ is the difference between the labeled and unlabeled batch sizes. The semi-supervised parameters (τ, λ, and μ) were tuned, providing a high degree of model flexibility.

We developed a vision transformer model with the base architecture of MobileViT [34], which uses automated feature engineering similar to standard CNNs [23]. MobileViT, which we also used in the “Temporal Neural Networks” section, is a transformer-based model that employs mechanisms of attention and depthwise dilated convolution. The model uses efficient convolution and transformer blocks, allowing for high efficiency and inference speed similar to the lightweight CNN used in our “Baseline Model” [12, 13]. We tested three different backbones for MobileViT (i.e., XXS, XS, and S), which varied in terms of the number of transformer layers, more sophisticated feature extraction, and number of parameters, allowing for an optimal trade-off between model size and performance. We developed our model using TensorFlow 2.0 and trained using a high-performance Google Cloud TPU.

Using the same StairNet data set split distribution as our baseline model [12, 13], we reduced the labeled training data from 461,328 to 200,000 images to study the impact of reduced annotations. To address the issue of unknown class distribution and image quality of the unlabeled data, we used our StairNet baseline model to retrieve the logits of the 4.5 million unlabeled images from ExoNet, which were thresholded using the FixMatch approach.

After processing the unlabeled data, 1.2 million images surpassed the 0.9 τ cutoff threshold. The resulting subset of images had a pseudo-label distribution that closely resembled the original StairNet data set [12, 13] (i.e., 5.5% for IS, 1% for IS–LG, 90.1% for LG, and 3.4% for LG–IS). The lightest MobileViT XXS model (900,000 parameters) was the fastest to train and infer among the three variants but had low accuracy during training. The balanced MobileViT XS model (1.9 million parameters) provided the best trade-off between compactness and performance. The largest MobileViT S model (4.9 million parameters) had the slowest training and inference times, while having worse overall performance likely due to overfitting.

During training, the data imbalance of the labeled and unlabeled data sets was handled by replacing standard cross-entropy with a focal loss class weight penalization of γ = 3 to penalize hard negatives. We also tested the exponential moving average (EMA), which smoothed the parameters and produced significantly better results than the final weight matrices without EMA. The resulting model showed good convergence and well-balanced performance across classes, but the overall image validation accuracy with focal loss was inferior to that of the previous vanilla cross-entropy loss experiments.

To reduce the number of false positives, augmentations were applied to the labeled training set, including minor translations, rotations, contrast, and saturation. We tested the L2 parameter loss and decoupled weight decay during training [46]. However, our best models did not include any weight decay regularization. We experimented with both cosine annealing schedule, as suggested in FixMatch [41], and cosine decay with restarts [47]. The former was found to be more resilient and consistent and thus was used in our final model. Several experiments were conducted to determine the optimal ratio of unlabeled to labeled data (μ) and the unsupervised loss weight parameter (λ).

Our semi-supervised learning model achieved classification accuracies of 99.2% and 98.9% on the StairNet training and validation sets, respectively. When evaluated on the test set, the model achieved an overall image classification accuracy of 98.8%, a weighted F1-score of 98.9%, a weighted precision value of 98.9%, and a weighted recall value of 98.8%. Similar to our “Baseline Model”, the two transition classes (LG–IS and IS–LG) achieved the lowest categorical accuracies (90.6% and 90.4%), which can be attributed to having the smallest class sizes. Overall, our semi-supervised learning model achieved a similar image classification performance as our “Baseline Model” [12, 13] but required 35% fewer labeled images, therein improving the training efficiency.

### Embedded deployment

Finally, we developed a pair of integrated smart glasses to move towards a more human-centred design [22]. One of the limitations of our previous models was their use of images from a chest-mounted smartphone camera. These images do not necessarily coincide with the user's visual field, and thus are more difficult to infer intent. However, previous head-mounted cameras [48–50] have mainly been limited to off-device inference using desktop computers and cloud computing. Prior to this study, an integrated system for visual perception of human–robot walking environments had not yet been designed, prototyped, and evaluated. This gap could be explained by limitations in embedded computing, which have only recently been alleviated by advances in hardware and deep learning model compression methods.

Consequently, we developed a novel pair of AI-powered smart glasses that uniquely integrate both sensing and computation for visual perception of human–robot walking environments while achieving high accuracy and low latency. We integrated the mechatronic components all within a single system, which is lightweight and has a small form factor so as not to obstruct mobility or user comfort. Computationally, it has sufficient memory and processing power for real-time computing with live video streams. Inspired by commercial smart glasses, such as Google Glass [48] and Ray-Ban Stores [49], our design includes a forward-facing camera aligned with the user’s field of view (i.e., egocentric), with a microcontroller for computational processing on the side of the glasses. This design allows for a slightly larger processor to support onboard inference without obstructing the visual field.

We used the ArduCam HM0360 VGA SPI camera due to its relatively high resolution, fast frame rate, and low power consumption (i.e., under 19.6 mW [51]). The camera’s frame rate of 60 fps should be fast enough to support robot control while providing sufficient resolution (680 × 480) to portray the environment, with an input size larger than most deep learning models. For embedded computation, we used the Raspberry Pi Pico W microcontroller due to its enhanced processing power, large memory, small form factor, and wireless communication. The Pico contains Dual ARM 133 MHz processors, 64 kB SRAM and 2 MB QSPI flash memory, and a small form factor of 21 mm × 51.3 mm, allowing for sufficient computation for model inference while easily integrating into eyeglass frames. The microcontroller can also wirelessly communicate and interface with external robotic devices and computers via a single-band 2.4 GHz Wi-Fi connection or through Bluetooth 5.2.

**Fig. 5:**
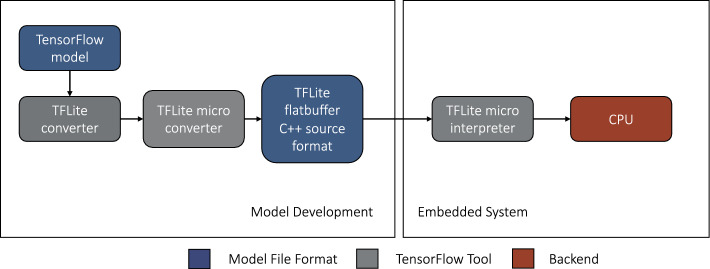
Model conversion and deployment pipeline for our smart glasses [22], which we developed to deploy and test our StairNet model for real-time embedded computing

**Fig. 6:**
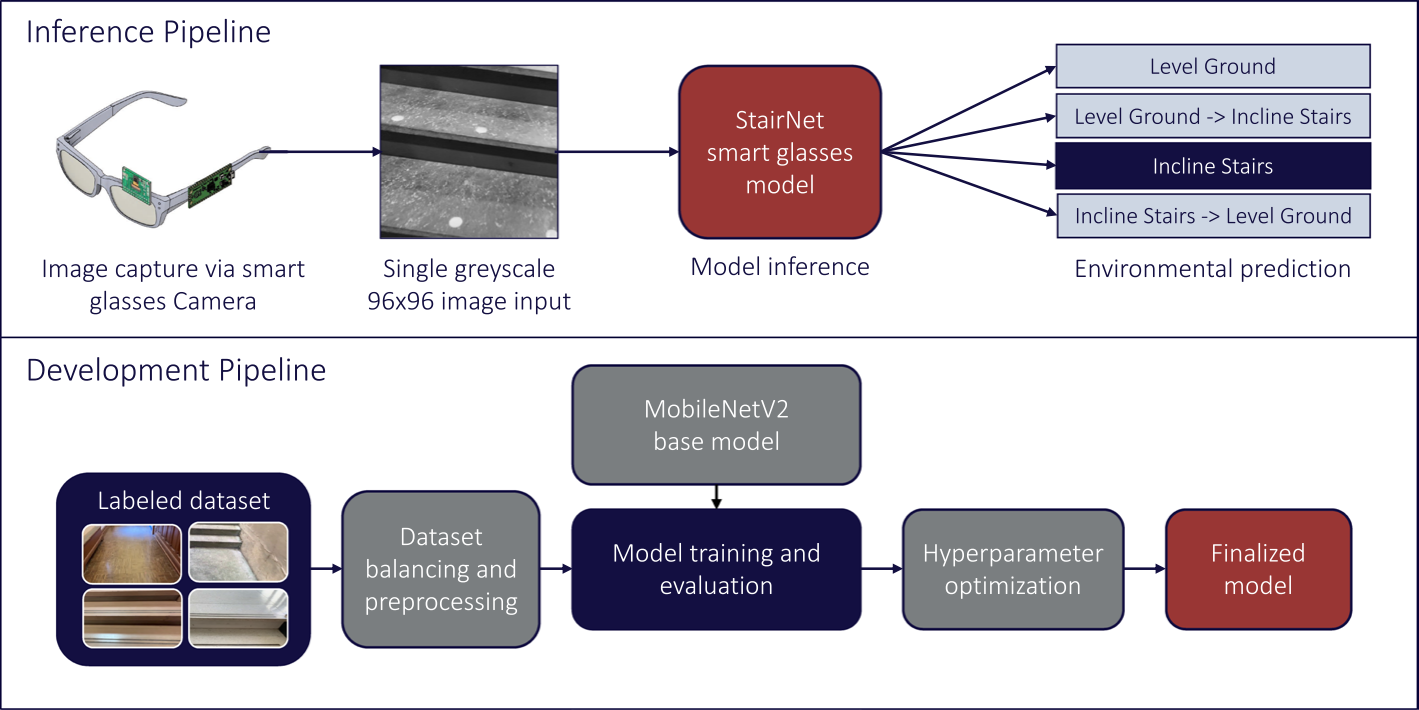
Inference and development pipelines for our smart glasses StairNet model trained using supervised learning and single images. Compared to our other models, the smart glasses performed stair recognition using a head-mounted camera and an embedded system

We developed a deep learning model using a similar approach as our “Baseline Model”. However, fine-tuning was required to convert the model from a chest-mounted domain to an eye-level domain. To do this, the baseline model was retrained using 7,250 images adapted from the Meta Ego4D data set [52] that we manually re-labelled, which contained walking environments that matched the StairNet classes (i.e., LG, LG–IS, IS, and IS–LG), with an input size of 96 × 96. We used the lightweight MobileNetV1 architecture to reduce the model size for embedded computing compared to larger architectures, such as MobileNetV2. We performed hyperparameter optimization for batch size and learning rate with optimal values of 32 and 0.0001, respectively. The final model contained 219,300 parameters, was converted to a TensorFlow Lite model using int8 quantization and further reduced to a TensorFlow Micro model for deployment (Figs. 5 and 6). We measured the embedded inference time as the loop of loading the most recent image captured and running the model inference on the microcontroller.

The average embedded inference speed was 1.47 s from reading the image to outputting the predicted label. Prior to fine-tuning, the model achieved a similar performance to our baseline StairNet model with 98.3% accuracy. With fine-tuning using the Ego4D images from head-mounted cameras, the model achieved 98.2% accuracy. To our knowledge, these AI-powered smart glasses are the first to integrate both sensing and deep learning computation for visual perception of human–robot walking environments.

## Discussion

In summary, here we present a comprehensive overview of StairNet, which we created to support the development of new deep learning models for visual perception of human–robot walking environments, with an emphasis on stairs. Our initiative places emphasis on lightweight and efficient neural networks for onboard real-time inference on mobile and embedded devices. First, we summarize the development of our StairNet data set with over 515,000 manually labeled images [12]. We then summarize and compare the performances of different algorithms (i.e., 2D and 3D CNN, hybrid CNN and LSTM, and ViT networks [12, 20, 21]), training methods (i.e., supervised learning with and without temporal data, and semi-supervised learning with unlabeled images [12, 20, 21]), and deployment methods (i.e., mobile and embedded computing [13, 22]) using the StairNet data set. Our models consistently achieved high classification accuracy (i.e., up to 98.8%) with different designs, offering trade-offs between model size and performance. When deployed on mobile devices with GPU and NPU accelerators, our deep learning models achieved inference speeds up to 2.8 ms [13]. When deployed on our custom-designed CPU-powered smart glasses, the inference speed was slower (i.e., 1.5 s) [22]. Overall, our results suggest that StairNet can serve as an effective platform to develop and study new deep learning models for visual perception of stair environments for human–robot walking, with intended future applications in environment-adaptive control of robotic prosthetic legs, exoskeletons, and other mobility assistive technologies.

Our StairNet models offer several benefits over other stair recognition systems [6–11, 14–17, 27]. Many studies have been limited to statistical pattern recognition and machine learning algorithms that require manual feature engineering. In contrast, our models use multilayer deep neural networks for automatic feature extraction, which has shown to be superior to hand-engineered features [23]. In addition, our models benefit from the high quantity and quality of the StairNet data set, with over 515,000 manually annotated images, allowing for more generalizable systems. Previous research has used smaller data sets (see Table 2). These differences can have important practical implications as machine learning typically requires large amounts of diverse data. The increased generalization potential of our models also eliminates the need for explicit requirements for the camera pose or angle, unlike past studies that relied on meticulous rule-based thresholds for the dimensions of the user and environments [10].

**Table 2:** Summary of vision-based stair recognition systems for robotic leg prostheses and exoskeletons

Reference	Camera	Position	Data set Size	Classifier	Computing Device	Test Accuracy
[11]	RGB	Waist	7284	Convolutional neural network	NVIDIA Titan X	99.6%
[10]	Depth	Chest	170	Heuristic thresholding and edge detector	Intel Core i5	98.8%
[9]	Depth	Leg	8455	Support vector machine	Intel Core i7-2640M	98.5%
StairNet	RGB	Chest	515,452	Convolutional neural network	Google Cloud TPU	98.4%
[17]	Depth	Leg	3000	Convolutional neural network	NVIDIA Quadro P400	96.8%
[8]	Depth	Leg	109,699	Cubic kernel support vector machine	Intel Core i7-2640M	95.6%
[14]	RGB	Chest	34,254	Convolutional neural network	NVIDIA TITAN Xp	94.9%
[15]	RGB	Head	123,979	Bayesian deep neural network	NVIDIA Jetson TX2	93.2%
[16]	RGB	Leg	123,954	Bayesian deep neural network	NVIDIA Jetson TX2	92.4%
(27)	RGB	Chest	542,868	Convolutional neural network	Google Cloud TPU	70.8%

The data set size (i.e., the number of images) and test accuracy are only for the environment classes relating to level-ground walking and stair ascent. The systems are organized in terms of the test accuracy (%)

As part of the StairNet initiative, we have studied a variety of deep learning models and training methods (Table 3), each of which offer unique advantages and trade-offs. For example, the MoViNet 3D CNN using temporal data [20], as described in the “Temporal Neural Networks” section, achieved the highest classification accuracy on our StairNet test set compared to our baseline 2D CNN model from the “Baseline Model” section, with a performance increase of 1.1%, demonstrating the benefit of temporal data for visual perception of human–robot walking environments. However, the model contains a relatively large number of parameters (4.03 million) and numerical operations (2.5 GFLOPs), which could hinder deployment and real-time inference on mobile and embedded devices with limited computational resources. These models might be better suited for use cases with access to reliable cloud computing. For model efficiency, our MobileViT XS model trained using semi-supervised learning in the “Semi-Supervised Learning” section achieved the highest NetScore of 202.4 [21], demonstrating the benefit of using lightweight vision transformers to reduce model parameter count compared to standard convolutional neural networks. In addition, our semi-supervised learning model improved training efficiency by reducing the number of required labelled images by 35% while maintaining similar image classification accuracy as our baseline StairNet model. The high efficiency of the MobileViT XS model makes it well-suited for our computer vision application.

**Table 3:** Summary of our StairNet stair recognition systems

Type	Data set size	Training approach	Architecture	Change in accuracy compared to baseline	NetScore	Model Parameters (millions)
Baseline Neural Network	515,452 labeled	SL—Single frame	MobileNetV2	0%	186.8	2.3
Temporal Neural Networks*	515,452 labeled	SL—M1	MoViNet	+ 1.1%	167.4	4.0
		SL—M1	MobileNetV2 + LSTM	+ 0.1%	132.1	6.1
		SL—M1	MobileViT-XXS + LSTM	− 0.2%	155.0	3.4
		SL—MM	MobileNetV2 + LSTM	− 26.5%	120.1	6.0
Semi-Supervised Neural Network	300,000 labeled, 900,000 unlabeled	SSL—Fix Match	MobileViT-XS	+ 0.4%	202.4	1.9
		SSL—Fix Match	MobileViT-XXS	− 0.7%	186.5	0.9
		SSL—Fix Match	MobileViT-S	− 1.2%	169.7	4.9

The models were evaluated based on image classification accuracy and efficiency (i.e., NetScore, where higher is better). The systems are organized by model type. We tested supervised learning (SL) and semi-supervised learning (SSL) methods, and many-to-one (M1) and many-to-many (MM) temporal neural networks. The data set sizes for our baseline and temporal neural networks were 515,452 labeled images, and 300,000 labeled images and 1.8 million unlabeled images for our semi-supervised learning networks

^*^Evaluated using the video-based train/validation/test split as described in the “Temporal Neural Networks” section

We also studied mobile and embedded computing through our development of a new mobile app [13] and smart glasses [22]. The mobile app uses a TFLite interpreter and on-device GPU and NPU accelerators. Inference speeds on the mobile app were as fast as 2.75 ms. We also developed a novel pair of fully integrated smart glasses with onboard sensing and deep learning computation. These glasses align with the user’s head orientation and visual field-of-view, therein having greater potential to infer intent. However, limitations in the embedded system yielded slower inference speeds of 1.5 s, presenting a trade-off between human-centered design and performance. Future research will focus on improving the embedded inference speed. Note that our past applications running on iOS devices were developed as examples to demonstrate the feasibility of mobile deployment. Our StairNet models run using TFLite, which is compatible with a wide variety of computing systems (e.g., desktop, cloud, mobile, and embedded) and are not limited to deployment on just the devices tested herein.

Despite this progress, our research still has several limitations. To evaluate performance, we used the StairNet test set. Although test sets are common practice in deep learning [23], the true real-world performance, generalizability, and application of our models was not analyzed in a deployed environment. In addition, during the development of our temporal models, we identified a limitation of the training method used for our baseline and semi-supervised models as the train/validation/test splits were performed randomly between images. This caused data leakage between the different data subsets, with unintentionally higher classification performances for our baseline and semi-supervised models. Retesting revealed an updated baseline accuracy of 97.2% when using data set splits with randomly sorted videos without neighboring frames in multiple data subsets. To address this, performance evaluations were made based on the change in accuracy compared to our baseline model of the respective test set. For future research using our StairNet data set, we recommend using the video-based training/validation/test splits.

It is worth mentioning that state-of-the-art machine learning models and methods are continuously being developed. For example, during the course of our development of the temporal models, research on transformers [53] and multilayer perceptrons [54] showed the ability to eliminate the need to process each frame for the encoder and temporal blocks separately by adapting the models to take 3D sequence inputs by modifying the patch-embedding block, which can significantly improve the efficiency in processing and inference. For our semi-supervised learning research, other algorithms besides FixMatch [41] could have also been used to further reduce the number of required labeled images, such as invariant semantic information clustering [55] and cross-level discrimination for unsupervised feature learning [56]. Our visual perception systems, especially the smart glasses, could also be extended to other applications such as providing sensory feedback to persons with visual impairments by leveraging recent advances in large language models [57].

We also want to emphasize that we designed our environment recognition systems to create the opportunity to improve the speed and accuracy of locomotion mode recognition by minimizing the search space of potential solutions based on the perceived walking environment. The intended future applications are environment-adaptive control of robotic prosthetic legs and exoskeletons, which were not studied here. However, the theoretical feasibility of this has been demonstrated by previous studies, such as Huang et al. [58], which found improvements in locomotion mode recognition by adding simulated environment data via Bayesian fusion. Our StairNet initiative builds on this approach via creating large-scale vision systems powered by deep learning that can accurately generalize across complex real-world environments.

In conclusion, the results of numerous experiments presented herein provide consistent evidence that StairNet can be an effective platform to develop and study new deep learning models for visual perception of human–robot walking environments, with an emphasis on stair recognition. This research aims to support the development of next-generation AI-powered control systems for robotic prosthetic legs, exoskeletons, and other mobility assistive technologies.

## Data Availability

The data set generated and analyzed during the current study is available in the IEEE Dataport repository, https://ieee-dataport.org/documents/stairnet-computer-vision-dataset-stair-recognition..

## Abbreviations

EMG: Electromyography
CNN: Convolutional neural network
TPU: Tensor processing unit
TFLite: TensorFlow Lite
CPU: Central processing unit
GPU: Graphics processing unit
NPU: Neural processing unit
LSTM: Long–short-term memory
NAS: Neural architecture search
SGD: Stochastic gradient descent
EMA: Exponential moving average
MM: Many-to-many
M1: Many-to-one
SSL: Semi-supervised learning
SL: Supervised learning
